# The Effect of Copper Addition on the Activity and Stability of Iron-Based CO_2_ Hydrogenation Catalysts

**DOI:** 10.3390/molecules22091579

**Published:** 2017-09-20

**Authors:** Matthew J. Bradley, Ramagopal Ananth, Heather D. Willauer, Jeffrey W. Baldwin, Dennis R. Hardy, Frederick W. Williams

**Affiliations:** 1 ASEE Postdoctoral Research Associate, Naval Research Laboratory, Materials Science and Technology Division, Washington, DC 20375, USA; matthew.bradley.ctr@nrl.navy.mil; 2 Naval Research Laboratory, Chemistry Division, Washington, DC 20375, USA; ramagopal.ananth@nrl.navy.mil (R.A.); fredsshadwell@gmail.com (F.W.W.); 3 Naval Research Laboratory, Materials Science and Technology Division, Washington, DC 20375, USA; 4 Naval Research Laboratory, Acoustics Division, Washington, DC 20375, USA; jeff.baldwin@nrl.navy.mil; 5 NOVA Research Inc., 1900 Elkin Street, Alexandria, VA 22308, USA; kashardy@gmail.com

**Keywords:** CO_2_ conversion, selectivity, activity, modelling, iron-based, copper

## Abstract

Iron-based CO_2_ catalysts have shown promise as a viable route to the production of olefins from CO_2_ and H_2_ gas. However, these catalysts can suffer from low conversion and high methane selectivity, as well as being particularly vulnerable to water produced during the reaction. In an effort to improve both the activity and durability of iron-based catalysts on an alumina support, copper (10–30%) has been added to the catalyst matrix. In this paper, the effects of copper addition on the catalyst activity and morphology are examined. The addition of 10% copper significantly increases the CO_2_ conversion, and decreases methane and carbon monoxide selectivity, without significantly altering the crystallinity and structure of the catalyst itself. The FeCu/K catalysts form an inverse spinel crystal phase that is independent of copper content and a metallic phase that increases in abundance with copper loading (>10% Cu). At higher loadings, copper separates from the iron oxide phase and produces metallic copper as shown by SEM-EDS. An addition of copper appears to increase the rate of the Fischer–Tropsch reaction step, as shown by modeling of the chemical kinetics and the inter- and intra-particle transport of mass and energy.

## 1. Introduction

The Department of Defense (DOD) is the single largest buyer and consumer of jet fuel in the world [1,2]. Currently, it is estimated that DOD purchases almost 3 billion gallons of jet fuel per year. Due to the instability in fuel prices and a foreseeable reduction in fossil fuel resources, the U.S. Navy has an interest in maintaining U.S. energy security long term by investing in new sources of fuel that are more environmentally friendly [3]. Generating jet fuel from environmental sources of carbon dioxide (CO_2_) and hydrogen (H_2_) at the point of use could lead to strategic advantages and potential long term cost savings [4]. A key part in successfully synthesizing jet fuel from these resources involves the development of stable and highly selective CO_2_ hydrogenation catalysts for thermochemical processes.

The abundance of CO_2_ in the atmosphere and its documented impact on climate change have led to a rapidly growing research effort associated with using it as a carbon source [4,5,6]. Carbon and hydrogen are the principle building blocks needed to synthesize hydrocarbons or alcohols [7] to be used as chemicals and/or fuel. However, thermochemical methods for synthesizing fuel from CO_2_ require overcoming a large energy barrier and the energetically costly electrolysis process used to produce H_2_ [6]. A review by Porosoff et al. [8] addresses the need to develop catalysts that increase the efficiency of hydrocarbon production as well as decrease undesirable side products, such as methane and carbon monoxide, from the product stream.

Due to a combination of factors including cost, abundance, and activity, iron-based catalysts have been identified as candidates for industrial CO_2_ hydrogenation. However, one challenge with iron-based CO_2_ hydrogenation catalysts is their vulnerability towards water produced during the reaction [9,10]. Unlike traditional Fischer–Tropsch reactions (FT), there are two moles of water produced for every one mole of CO_2_ consumed [11,12,13]. Water is known to oxidize active sites, hydrogenate supports, and sinter catalyst sites [14,15,16,17,18]. In the past, efforts have been made to protect the catalyst by adding a start-up solvent or using a gas recycling system that sufficiently removes the water from the gas on each cycle [19,20]. An additional route to increasing a catalyst’s resistance to water is to add elements that are more resistant to oxidation. There has been some promising data that adding copper in small amounts to a supported iron catalyst can drastically improve activity [21,22,23]. Some modeling efforts of CO_2_ hydrogenation on the iron copper intermetallic (100) phase support the hypothesis that small amounts of copper will be beneficial to the system [24]. There is also extensive work on how catalysts change as a result of exposure to syngas conditions [25,26,27,28,29], but there is very little characterization work being done on FeCu/K catalysts in CO_2_ hydrogenation conditions.

In response to this gap in the literature, we report on the activity, composition, morphology, and kinetics of adding copper to well-characterized iron-based catalysts with the aim of optimizing a new, more efficient CO_2_ hydrogenation catalyst in this paper. Additionally, a detailed model for the fixed-bed reactor was developed to derive and understand the kinetics governing the activity and selectivity of the iron-based catalyst-containing copper metal. The model considers mass transfer between the catalyst particles and within a given particle. The species diffuse into individual particles and react inside the pores, and exchange mass with fluid flowing within the interstitial space of the particle bed [30,31,32]. The model also considers heat transfer associated with exothermic reactions occurring in the particles in the fixed-bed. The fluid mixture and packed bed properties depend on temperature, composition, and porosity. The model predictions are compared to the experimental data for the catalyst-containing copper metal.

## 2. Results

To evaluate the potential benefits of adding copper to an iron-based CO_2_ reduction catalyst, a standard iron CO_2_ hydrogenation catalyst that has been covered extensively in the literature was used as the baseline catalyst matrix [6,9,31,33,34].

Table 1 shows that a pure iron catalyst (100Fe/K) has similar activity to what has been previously published for studies performed at 300 °C and 290 psi [6,9,31,32,33]. The overall CO_2_ conversion was 24%, 79% of which was converted into useful hydrocarbons. Methane (5%) and carbon monoxide (16%) made up the rest of the product stream. This leads to an overall hydrocarbon yield of 19%. Hydrocarbon yield is defined as the percent of carbon atoms that fully convert into a C2+ hydrocarbon product. Anderson–Schultz–Flory (ASF) growth distribution plots were also used to evaluate the length distribution of the hydrocarbon chains. The chain growth probability value (*α*) for 100Fe/K is 0.98, which is a measure of the slope of ln(WN/N) vs. N, where N is the carbon number and WN is the weight fraction of hydrocarbons containing N carbons. The last measure used to evaluate the activity of the catalysts was the olefin/paraffin (O/P) ratio, which is simply the number of olefins products divided by the number of paraffin products. Since olefins are much easier to perform subsequent chemistry upon, higher O/P is more desirable. The O/P ratio for 100Fe/K was 4.3.

Copper was then added to the catalyst at various ratios. Iron was replaced by 10% (90Fe10Cu/K), 20% (80Fe20Cu/K), and 30% (70Fe30Cu/K) copper while maintaining the same amount of potassium and alumina support. The total amount of active metal species (iron and copper) did not change between catalysts. Only the ratio of iron and copper was altered. In all cases, there was an increase in catalyst activity under the same conditions (300 °C and 290 psi). The 90Fe10Cu/K catalyst showed the best activity, almost doubling the CO_2_ conversion (41%) and increasing hydrocarbon selectivity (88%). It also increased the O/P ratio to 4.9. As the copper:iron ratio was increased from 10 to 20%, CO_2_ conversion decreased from 41 to 30% and hydrocarbon yield decreased in favor of the formation of more methane and CO. Further increases in copper:iron ratio yielded an increase in CO_2_ conversion to 36% and similar activity and selectivity as shown for the 90Fe10Cu/K catalyst. The increase in hydrocarbons in these FeCu/K samples seems to correlate with a decrease in CO selectivity. Therefore, more intermediate CO is being converted into full hydrocarbons, which would suggest an increase in the rate of the FT step of the reaction. The only disadvantage from the addition of copper to the iron catalyst was a slight decrease in Anderson–Shultz–Flory chain growth probability values *α* (0.98 vs. 0.90). The result of the increase in CO_2_ conversion and hydrocarbon selectivity leads to a huge increase in the hydrocarbon yield. The 90Fe10Cu/K catalyst had the highest yield of 36%, 70Fe30Cu/K had the second highest yield of 32%, and 80Fe20Cu/K had the worst yield of the FeCu/K catalysts at 25%. The discrepancy in CO_2_ conversion and catalyst selectivity found for 80Fe20Cu/K will be substantiated further in this paper.

Table 2 provides preliminary results that support literature findings on the necessity of adding potassium to the catalyst matrix to achieve higher catalyst activity [24,32,33]. Without the addition of potassium to the catalyst matrix, CO_2_ conversion drops to 17% and both the methane (35%) and carbon monoxide (11%) selectivities increase (Table 2). In addition, the hydrocarbon selectivity decreases to 54% and the hydrocarbons formed are completely saturated. Since no olefins were formed from catalysts without potassium present in the catalyst matrix, the O/P ratio was zero. This further highlights the role potassium has on iron-based catalyst activity.

In order to determine whether the FeCu oxide phase was critical to the observed increase in activity, a sample was tested that consisted of a physically mixed 100Fe/K catalyst and 100Cu/K catalyst in the same ratio as the 90Fe10Cu/K and the results were compared (Table 3). This physically mixed sample contained the same amount of all the elements in the previous samples but did not share any effects due to copper and iron being on the same supported substrate. The physically mixed sample showed a drop in CO_2_ conversion of 18%, less than 50% of what the 90Fe10Cu/K sample displayed. Methane selectivity increased to 14%, and CO selectivity dropped to 3%. No olefins were produced in the mixed sample, and the *α* value dropped from 0.95 to 0.75. With such a drastic change in activity, it is clear that the Fe and Cu reagents must have some cooperative effect when they are precipitated on the same support. To determine if a simultaneous deposition of Cu and Fe was necessary for this synergistic effect, a “layered” catalyst containing Fe and Cu was also synthesized in a step-wise fashion. In the “layered” sample, the iron and potassium portions were nucleated onto the support and calcined, then the copper solution was added, dried, and calcined (Table 3). No significant differences were observed between the co-precipitation and sequentially precipitated samples beyond an increase in O/P ratio.

Temperature effects were also studied to determine the kinetic parameters of a Fe80Cu20/K catalyst. Table 4 shows that at 250 °C, the CO_2_ conversion drops to a third of its value at 300 °C. When the temperature is increased to 340 °C, the conversion increases as well. Carbon monoxide selectivity drops below the detection limit at the lower temperature, but at a higher temperature there is only a minor increase in CO and methane. The loss in activity at low temperature is consistent with that reported by Pendyala et al. [35] due to increased water oxidation of the iron catalyst during the Fischer Tropsch Synthesis (FTS) step.

## 3. Discussion

### 3.1. Catalyst Characterization

**Figure 1:**
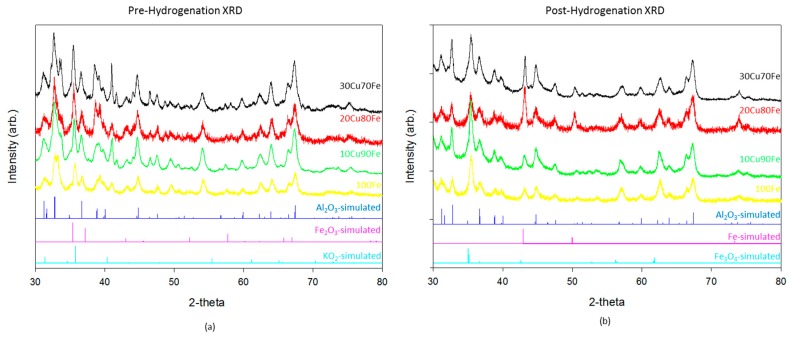
(**a**) Powder X-ray diffraction patterns of 100Fe/K (orange), 90Fe10Cu/K (grey), 80Fe20Cu/K (light blue), and 70Fe30Cu/K (dark blue) after calcination but before reduction and CO_2_ hydrogenation. For comparison, simulated patterns for *γ*-Al_2_O_3_ (yellow), KO_2_ (blue), and Fe_2_O_3_ (green) are also shown; (**b**) Powder X-ray diffraction patterns of 100Fe/K (grey), 90Fe10Cu/K (light blue), 80Fe20Cu/K (orange), and 70Fe30Cu/K (dark blue) after CO_2_ hydrogenation. For comparison, simulated patterns for *γ*-Al_2_O_3_ (yellow), Fe (blue), and Fe_3_O_4_ (green) are also shown.

**Figure 2:**
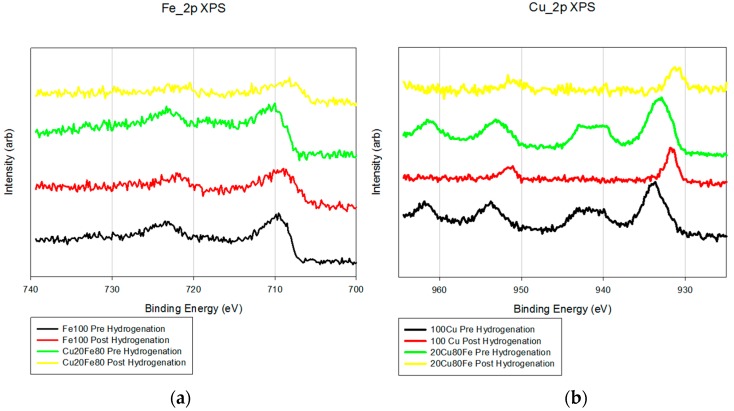
X-ray photoelectron spectroscopy data for both (**a**) Fe_2p and (**b**) Cu_2p.

Catalysts were characterized by XRD (Figure 1) and XPS (Figure 2) both before and after hydrogenation. There is relatively little difference in the XRD patterns between the different catalysts. The majority of the reflections in these samples both before and after hydrogenation are accounted for by gamma-Al_2_O_3_, the support material. The pre-hydrogenation catalysts also show a fully oxidized Fe_2_O_3_ phase and KO_2_ phase (Figure 1a). Although K_2_O is the standard phase of potassium oxide, we consistently observe the KO_2_ phase, which is indicative of how the potassium arranges itself in the catalyst matrix.

After reduction and subsequent CO_2_ hydrogenation (Figure 1b), the Fe_2_O_3_ phase shifts to the reduced Fe_3_O_4_ inverse spinel phase, and in samples with a higher copper ratio some metallic face center cubic (FCC) phase begins to form as well. It is important to note that, although the crystal phases used for simulation are Fe-based, the reality is that they are likely Fe–Cu intermetallic or alloy phases. The small difference in atomic radius between the two atoms and the complex nature of the catalyst make differentiation of those phases difficult in this system. As the Cu ratio is increased, the metallic FCC phase also increases. One possible explanation is that the higher reduction potential of Cu leads to an increase in the metallic phase in the reductive environment. Although some reduction should be expected due to the presence of H_2_, there does not appear to be a correlation between the increase in the FCC phase and an increase in catalytic activity for CO_2_ hydrogenation. This result differs from the modeling [24,25,26,27,28,29] precedent, which uses the metallic FCC phase as a catalytically active site in Fe_x_Cu_y_ catalyst. In the highest performing catalyst, 90Fe10Cu/K, there is no evidence of the metallic phase. It is only when the copper, which has a much larger reduction potential than iron, ratio increases that the oxide transitions reduces fully to the metal.

XPS also shows little difference between the Fe/K and FeCu/K samples (Figure 2). The Fe_2p analysis shows a minor shift (2–3 eV) toward a more reductive environment with the addition of copper. The Cu_2p pre-reduction samples show peaks at 943 eV that are due to the presence of Cu^2+^. After reduction, these peaks disappear and the peaks at 934 eV shift 3–4 eV, which is a more reduced form of copper.

**Figure 3:**
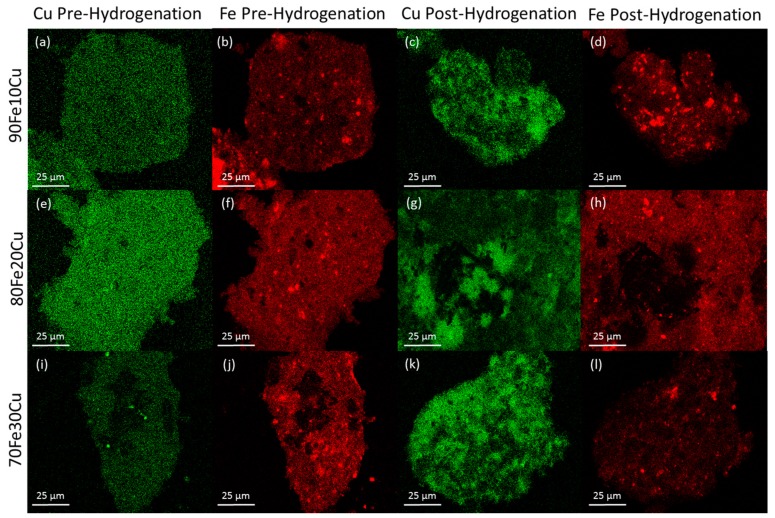
SEM-EDS mapping data where copper is labeled green and iron is labeled blue. (**a**) 90Fe10Cu/K copper map pre-hydrogenation; (**b**) 90Fe10Cu/K iron map pre-hydrogenation; (**c**) 90Fe10Cu/K copper map post-hydrogenation; (**d**) 90Fe10Cu/K iron map post-hydrogenation; (**e**) 80Fe20Cu/K copper map pre-hydrogenation; (**f**) 80Fe20Cu/K iron map pre-hydrogenation; (**g**) 80Fe20Cu/K copper map post-hydrogenation; (**h**) 80Fe20Cu/K iron map post-hydrogenation; (**i**) 70Fe30Cu/K copper map pre-hydrogenation; (**j**) 70Fe30Cu/K iron map pre-hydrogenation; (**k**) 70Fe30Cu/K copper map post-hydrogenation; and (**l**) 70Fe30Cu/K iron map post-hydrogenation.

The morphology of the system was examined by SEM-EDS. The analysis was performed on the catalyst samples both before and after hydrogenation (Figure 3). All samples observed before hydrogenation showed even distributions of copper and iron across the sample. However, in the post-hydrogenation samples, as the concentration of copper increases, the phase separation becomes evident. The 90Fe10Cu/K post-hydrogenation sample still shows an evenly distributed amount of Fe and Cu across the particle, with some copper-rich and iron-rich areas. The 80Fe20Cu/K post-hydrogenation sample shows the appearance of large copper-rich areas where almost no iron can be found. As the samples reach peak copper concentration in the 70Fe30Cu/K catalyst, we again see an even distribution of copper, but the copper signal is much stronger than that of the iron sample, implying that there is much more copper at the surface of the particle than there is iron. The most likely explanation of this would be a copper-rich layer coating the iron oxide bulk. This observation along with the XRD results is likely due to the immiscibility between metallic iron and metallic copper. It is hypothesized that copper is only miscible in the Fe_3_O_4_ inverse spinel phase to around 10%, and then begins to migrate out of the lattice under the conditions of CO_2_ hydrogenation [34,36]. Once on the surface, it would be much easier to reduce the individual copper atoms and metallic copper would form. Once enough metallic copper forms (around the 30% mark), it would begin to coat the surface entirely, suppressing the EDS signal for iron.

### 3.2. Computational Modeling

**Figure 4:**
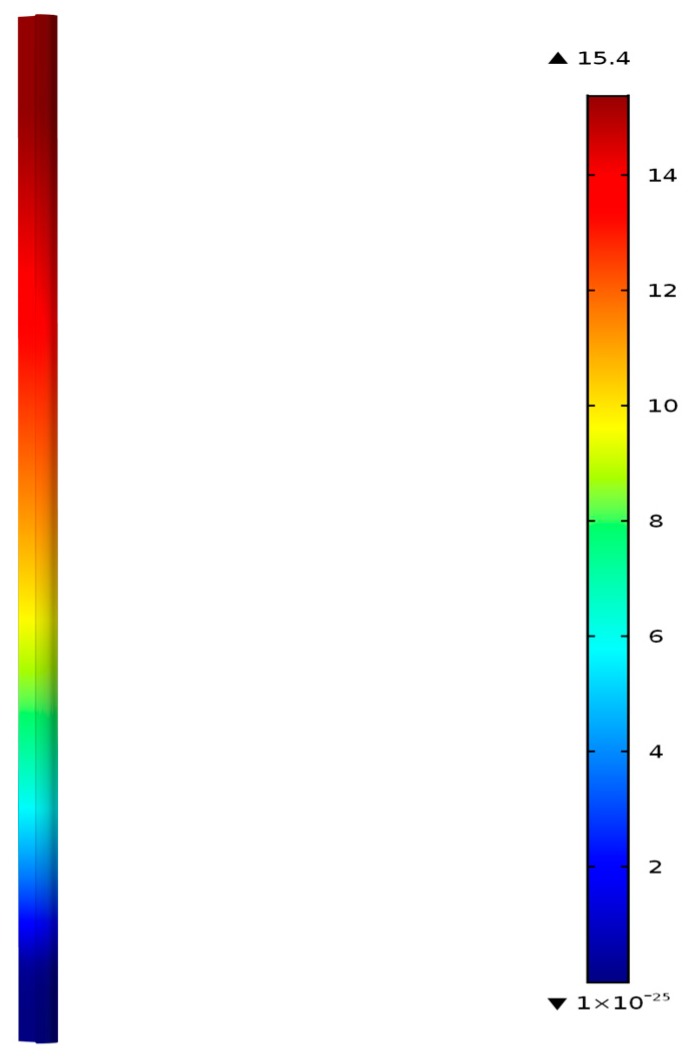
Reactor geometry with model-predicted contours of C_3_H_6_ concentration, mol/m^3^, formed by carbon dioxide hydrogenation in the catalyst bed (20Cu80Fe/K) with bed porosity of 0.51 and particle porosity of 0.38 at 300 °C and 20 bar total pressure at the reactor outlet.

A three-dimensional, non-isothermal, fixed-bed reactor model was developed by solving energy and convective-diffusion equations, which are coupled with Navier–Stokes equations for gas flow in a porous media, using a multiphysics modeling software, (COMSOL Inc., Burlington, VT, USA). The catalyst bed consists of particles, which are represented by a sub-grid model (Reactive Pellet Model) available in COMSOL. The sub-grid model describes the diffusion and reactions occurring inside a particle. The Millington–Quirk model is used to calculate the diffusivities of species inside a particle as functions of particle porosity. The surface area (22 m^2^/g) and pore volume (0.1638 mL/g) were determined via BET analysis. The species concentrations outside the particles (within the catalyst bed) are influenced by the chemical reactions occurring inside the particles. The sub-grid model was coupled to the fixed-bed model through a boundary condition that exchanges mass between gas outside the particle at a given location in the bed and inside the particle volume. Figure 4 shows the fixed-bed reactor geometry and the contours of C_3_H_6_ concentration in mol/m^3^ at 300 °C predicted by the model. The model considers only the rate controlling steps, and includes the formation of propene and methane, where the propene represents all the saturated and unsaturated hydrocarbon species (C2–C5+) measured in the experiments following Riedel et al. [30]. The thermodynamic and transport properties of each species are calculated using the CHEMKIN database as functions of temperature and composition. The mixture properties are calculated from the species properties based on local composition. The reactor is identical to the one employed in the experiments, which is a 0.0094 m inner diameter tube of 0.305 m length. It has inlet and outlet sections (0.0254 m each) and a middle section, which is filled with 20 g catalyst containing (FeCu)/K/Aumina 16M:12K:100Al_2_O_3_ by weight. In calculating the gas hourly space velocity (GHSV), only the total metal weight, mcat, 2.19 g was used. Figure 4 shows zero C_3_H_6_ concentration near the entrance of the catalyst bed, and the concentration increases from the inlet to the outlet of the reactor as the FT reactions forming the hydrocarbons continue to occur along the entire bed.

The steady-state, three-dimensional, convective-diffusion equations are given by

(1) $$\nabla \cdot \left(-D_{j}\cdot C_{j}\right)+u\cdot \nabla C_{j}=0,$$

where *D_j_* is the diffusion coefficient of species j in hydrogen, which is the dominant species in the gas mixture. Hydrogen is chosen to represent the mixture for calculating pseudo-binary diffusion coefficients. *C_j_* is the concentration of species *j*. Equation (1) describes the mass transport occurring within the interstitial space of the fixed-bed reactor. Equation (1) does not include diffusion and chemical reactions occurring inside a particle, which will be considered in a sub-grid model. The velocity vector, u, is obtained by solving the Navier–Stokes equations, which are given by

(2) ρ∇·u=0

and

(3) $$\rho \left(u\cdot \nabla \right)u=\nabla \cdot \left[-pI+\mu \left(\left(\nabla u\right)+{\left(\nabla u\right)}^{T}\right)\right]$$

where *ρ*, *μ*, and *p* are the density, dynamic viscosity, and pressure of the gas mixture, respectively. *I* is the identity vector, and the superscript “*T*” represents transpose. Equations (1)–(3) are applied for the entire reactor, including the inlet and outlet sections, where a free flow of gases occurs. For the fixed-bed in the middle section of the reactor, the momentum equation (Darcy–Brinkman) for the porous media is given by

(4) $$\frac{\rho }{\varepsilon_{p}}\left(u\cdot \nabla \right)\frac{u}{\varepsilon_{p}}=\nabla \cdot \left[-pI+\frac{\mu }{\varepsilon_{p}}\left(\left(\nabla u\right)+{\left(\nabla u\right)}^{T}\right)-\frac{2\mu }{3\varepsilon_{p}}\left(\nabla \cdot u\right)I\right]-\left(\mu K^{-1}+\frac{\rho }{\varepsilon_{p}^{2}}\left(\nabla \cdot u\right)\right)u.$$

(5) $$\varepsilon_{p}=1-\frac{\rho_{bed}}{\rho_{support}}$$

Here, *ε_p_* and *K* are the porosity and isotropic permeability of the fixed-bed, respectively. The gases flow within the interstitial volume of the bed. The porosity of the bed, *ε_p_*, was set at 0.51, and the permeability, *K*, was set at 5 × 10^−12^ m^2^. The porosity was calculated from Equation (5), where *ρ_support_* (2260.0 kg/m^3^) and *ρ_bed_* (1105 kg/m^3^) are the bulk density of the alumina support and the density of the catalyst bed, respectively. The density, *ρ_bed_*, of the catalyst bed is given by the mass of the bed (0.02 kg) divided by the volume of the bed, *V_reactor_* (1.81 × 10^−5^ m^3^).

The fixed-bed is assumed to be made of mono-dispersed spherical particles containing metal catalyst and having radius, *r_pr_* (250 μm). An individual particle has a porosity of *ε_pr_*, wherein the gases can diffuse but the convective flow within the micro-sized pores of the particle is neglected. The species concentration, *C_j_*, at a given position within the bed is influenced by the diffusion and chemical reactions occurring inside the particles. A sub-grid model equation describing the species concentration, *C*_*pr*,*j*_, distributions inside the pores of a spherical particle is given by

(6) $$\left[3V_{reactor}\varepsilon_{p}/r_{pr}^{3}\right]\left[r^{2}r_{pr}^{2}\varepsilon_{pr}\frac{\partial C_{pr,j}}{\partial t}+\nabla \cdot \left(-r^{2}D_{pr,j}\nabla C_{pr,j}\right)=r^{2}r_{pr}^{2}R_{pr,j}\right]$$

where *r* is the radial distance within a spherical particle and the diffusion coefficients, *D*_*pr*,*j*_, are given by

(7) $$D_{pr,j}=\varepsilon_{pr}^{4/3}D_{j}$$

where the particle porosity is given by

(8) $$\varepsilon_{pr}=1-\frac{\rho_{support}}{\rho_{solid}}.$$

Here, *ρ_solid_* (3650 kg/m^3^) is the solid material density of alumina and is higher than the bulk density, *ρ_support_*, of the particles. The particle porosity, *ε_pr_*, is calculated to be 0.38, which when divided by *ρ_support_* gives a pore volume of 0.1681 mL/g. The calculated pore volume of 0.1681 mL/g is close to the measured BET value of 0.1638 mL/g.

The gases enter the reactor at ambient temperature and get heated because the reactor walls are maintained at a constant temperature *T_w_*. The energy equation describes the temperature, *T*, distribution inside the fixed-bed reactor, and is given by

(9) $${\left(\rho C_{p}\right)}_{eff}u\cdot \nabla T+\nabla \cdot q=\underset{j}{\sum }R_{pr,j}h_{j}$$

where

(10) $${\left(\rho C_{p}\right)}_{eff}=\varepsilon_{total}\rho C_{p}+\left(1-\varepsilon_{total}\right)\rho_{solid}C_{p,solid}$$

the heat flux, *q*, is

(11) $$q=-k_{eff}\nabla T,$$

the effective thermal conductivity is

(12) $$k_{eff}=\varepsilon_{total}k+\left(1-\varepsilon_{total}\right)k_{solid},$$

the total porosity of the bed is

(13) $$\varepsilon_{total}=1-\frac{\rho_{bed}}{\rho_{solid}},$$

where the thermal conductivity, *k_solid_*, and specific heat, *C*_*p*,*solid*_, of the solid support material are 18 W/mK and 880 J/kg·K, respectively. The enthalpies of species, *h_j_*, are obtained from the CHEMKIN database. The ideal gas law was also used. The flow rates and composition of reactants at the inlet and the pressure at the outlet were specified as boundary conditions.

The reaction rates, *R*_*pr*,*j*_, are given by

(14) $$R_{pr,CO2}=-kSH\left(CO_{2}\times H_{2}-\left(\frac{CO\times H_{2}O}{K_{eq}}\right)\right),$$

(15) $$R_{pr,H2}=-2kSH\left(CO_{2}\times H_{2}-\left(\frac{CO\times H_{2}O}{K_{eq}}\right)\right)-2kFT\times CO\times H_{2}-3\times kFTs\times CO\times H_{2},$$

(16) $$R_{pr,H2O}=kSH\left(CO_{2}\times H_{2}-\left(\frac{CO\times H_{2}O}{K_{eq}}\right)\right)+kFT\times CO\times H_{2}+kFTs\times CO\times H_{2},$$

(17) $$R_{pr,CO}=kSH\left(CO_{2}\times H_{2}-\left(\frac{CO\times H_{2}O}{K_{eq}}\right)\right)-kFT\times CO\times H_{2}-kFTs\times CO\times H_{2},$$

(18) $$R_{pr,C3H6}=kFT\times CO\times H_{2},$$

where the kinetic constants are given by

(19) $$kSH=\frac{\rho_{cat}R_{g}Tk_{SH0}exp\left[-E_{SH}/R_{g}T\right]}{\left[\left(CO\right)+a_{SHH2O}\left(H_{2}O\right)+b_{SHCO2}\left(CO_{2}\right)\right]},$$

(20) $$kFT=\frac{\rho_{cat}R_{g}Tk_{FT0}exp\left[-E_{FT}/R_{g}T\right]}{\left[\left(CO\right)+a_{FTH2O}\left(H_{2}O\right)+b_{FTCO2}\left(CO_{2}\right)\right]},$$

(21) $$kFTs=\frac{\rho_{cat}R_{g}Tk_{FTs0}exp\left[-E_{FTs}/R_{g}T\right]}{\left[\left(CO\right)+a_{FTsH2O}\left(H_{2}O\right)+b_{FTsCO2}\left(CO_{2}\right)\right]},$$

(22) $$K_{eq}=K_{eq0}10^{\left(2.029-\frac{2073}{T}\right)},$$

(23) $$\rho_{cat}=m_{cat}/V_{reactor}.$$

The gas hourly space velocity, GHSV, is given by

(24) $$GHSV=F_{total}/m_{cat}.$$

In this study, GHSV is defined by Equation (24), where *F_total_* is the sum of the hydrogen and CO_2_ volumetric flow rates entering the reactor at standard conditions. Here, *m_cat_* is the weight (2.19 g) of the total elemental metal content in the catalyst without the support material. *V_reactor_* is the volume of the middle section of the reactor containing the fixed catalyst bed, and *T* is its temperature. *R_g_* is the gas constant. The species concentrations are represented by H_2_, CO_2_, CO, H_2_O, C_3_H_6_, and CH_4_. Propene is used as the representative species for all the hydrocarbons (C2–C5+) produced in the experiments with the exception of CH_4_, following the work of Riedel et al. [31]. The values for the kinetic parameters used in the model are provided in Table 5.

The CO, CH_4_, and C2–C5+ yields and selectivities are defined based on atomic carbon rather than on a molar basis. For example, the molar concentration of propene is multiplied by three in calculating its yield and selectivity because it contains three atomic carbons. As usual, CO_2_ conversion is the reacted CO_2_ as a percent of CO_2_ in the feed stream, and product selectivity is calculated as a percent of reacted CO_2_. Multiplying the conversion with the selectivity of the product gives the yield.

**Figure 5:**
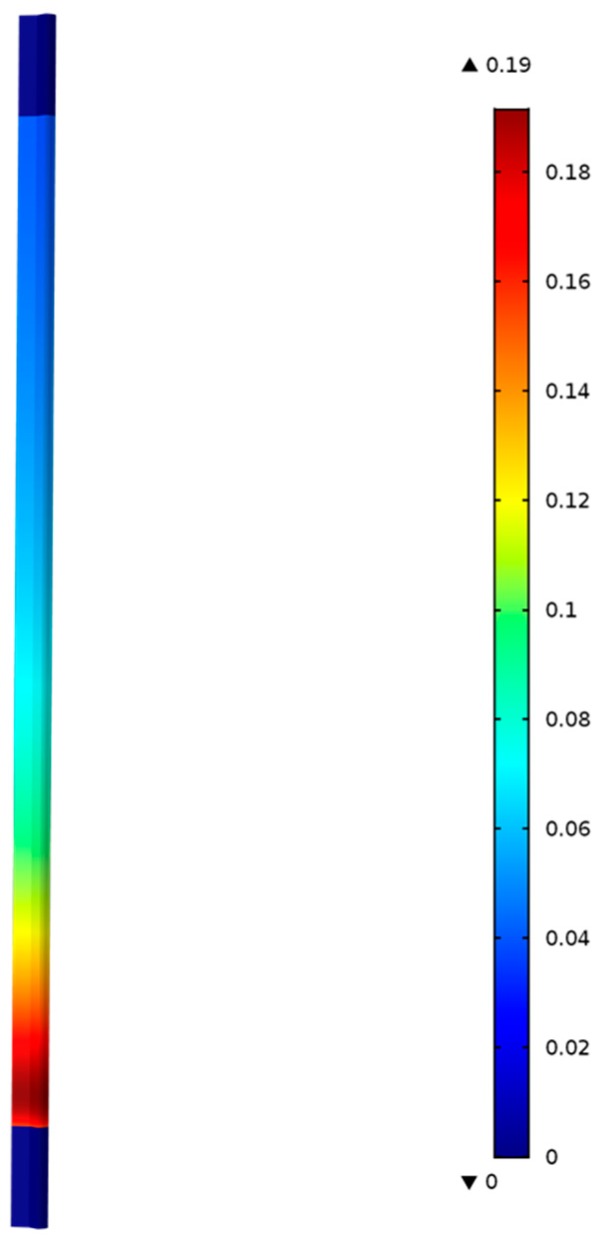
Contours of propene formation rate mol/(m^3^ s), RC_3_H_6_, at 300 °C and 20 bar total pressure.

**Figure 6:**
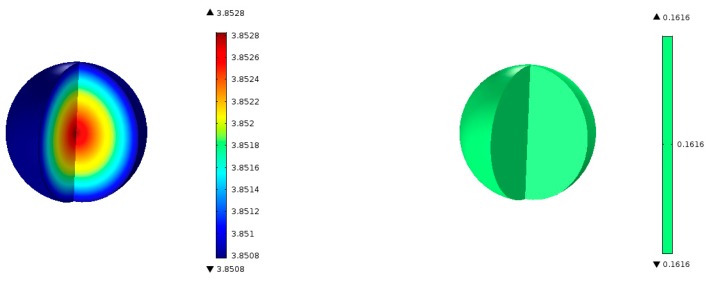
Contours of C_3_H_6_ concentration (mol/m^3^) and formation rate, RC_3_H_6_ (mol/(m^3^ s)), inside a 250 µm radius spherical catalyst pellet (80Fe20Cu/K) at 50 mm from the inlet show very small gradients due to high diffusion coefficients of the gas species at 300 °C and 20 bar total pressure.

**Figure 7:**
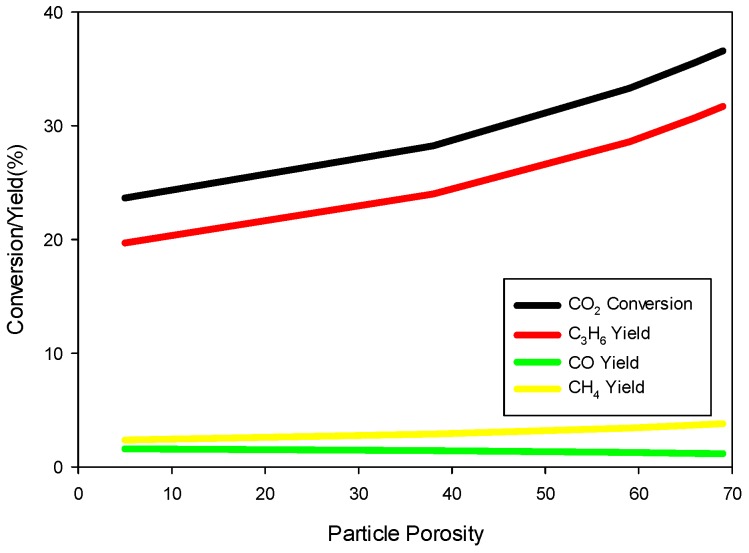
Effects of increased particle porosity and decreased bed porosity (with total porosity fixed at 69.7%) on percent CO_2_ conversion (black), and percent C_3_H_6_ (red), CH_4_ (yellow), and CO (green) at the reactor outlet as predicted by the model for a fixed 20 g of 80Fe20Cu/K catalyst at 300 °C and 20 bar total pressure.

**Figure 8:**
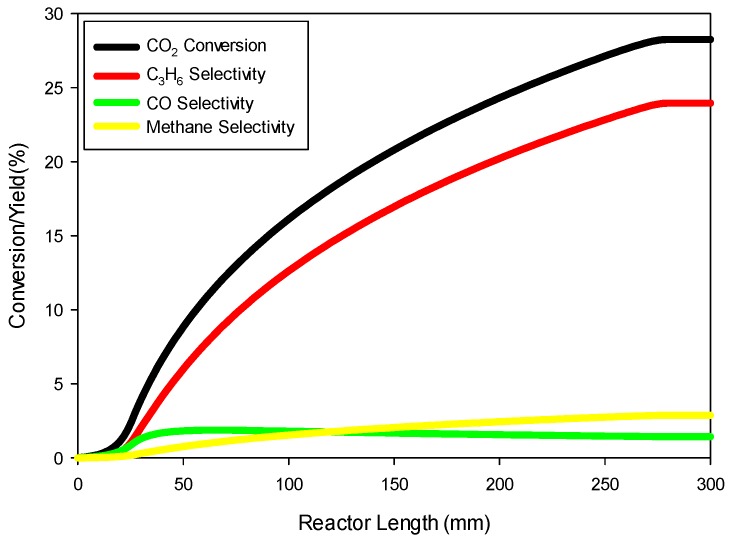
Profiles of percent CO_2_ conversion (black), and percent C_3_H_6_ (red), CH_4_ (yellow), and CO (green) yields along the length of the catalyst bed as predicted by the model for 20Cu/80Fe/K catalyst at 300 °C and 20 bar total pressure.

**Figure 9:**
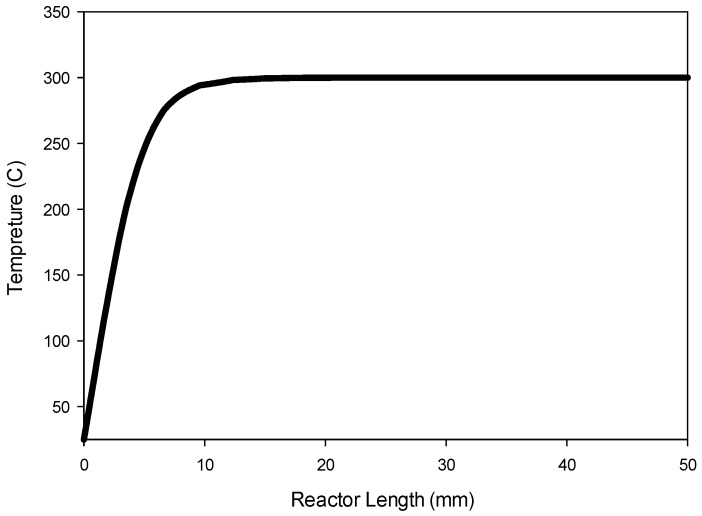
Profiles of temperature (°C) along the length (center axis) of the catalyst bed as predicted by the model for 80Fe20Cu/K catalyst at 20 bar total pressure and 300 °C imposed on the reactor wall.

The transient model Equations (1)–(24) are solved for 1000 s until steady-state solutions are achieved. Figure 5 shows the model predictions for the contours of propene concentration in the fixed-bed reactor for the reactor conditions, reactant flows (GHSV), and catalyst compositions used in the experiments as discussed in the previous section. Figure 5 shows the predicted contours of C_3_H_6_ formation rate (<0.19 mol/(m^3^ s)) due to the hydrogentaion reactions, and it shows a decreasing rate with distance along the reactor length, unlike the C_3_H_6_ concentration contours shown in Figure 4, which exhibit the opposite trend as one might expect. Figure 6 shows the predicted contours of C_3_H_6_ concentration and the rate of formation within a single catalyst pellet at 5 cm from the entrance of the reactor. The C_3_H_6_ concentration changes only very slightly across the radius of the spherical pellet because of a relatively fast diffusion of the gaseous species in and out of the pellet. The C_3_H_6_ formation rate predicted by the model is uniform across the pellet. We performed simulations for different particle sizes, and found that the uniform profiles for C_3_H_6_ hold for particle sizes up to 1 cm in diameter. So, it is clear that the model predictions are not very sensitive to the particle size. However, conversion and yields are sensitive to particle porosity despite fast diffusion within a particle. We increased the particle porosity with a total porosity ($\varepsilon_{total}$) fixed at 69.7% and a fixed catalyst loading such that the bed porosity decreases correspondingly. As the particle porosity is increased, more surface area becomes available for chemical reactions, affecting the conversion and yields as shown in Figure 7. Figure 8 shows the profiles of conversion and product yields along the length of the reactor for the 20Cu/80Fe/K catalyst bed. Our simulations show that the species concentrations inside catalyst pellets also follow almost identical profiles, as shown in Figure 8, because of negligible gradients across the catalyst pellet. Figure 9 shows the temperature profile along the center line of the reactor. Figure 9 indicates that the radial heat transfer is fast enough that the gases are heated very quickly from ambient temperature to the reactor wall temperature prior to entering the catalyst bed.

**Figure 10:**
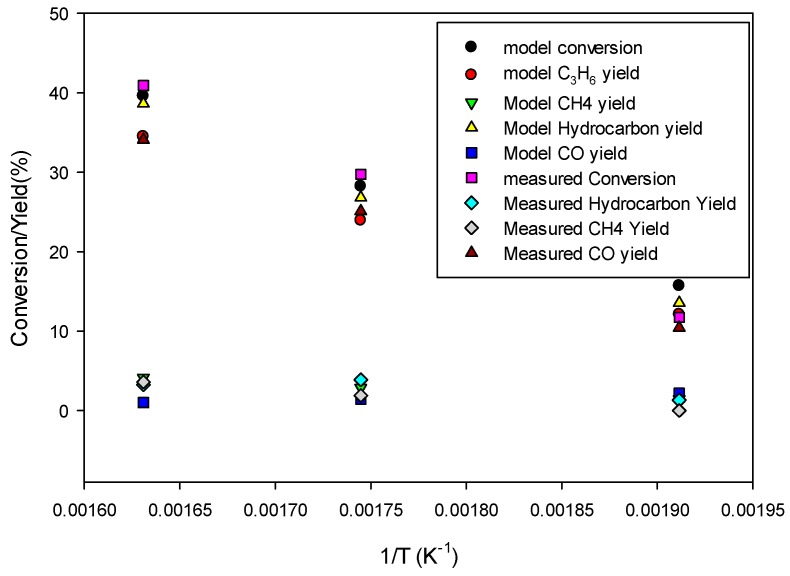
Comparison of model predictions with experimental data as functions of inverse temperature for 80Fe20Cu/K catalyst at reactor wall temperatures of 250 °C, 300 °C, and 340 °C and a gas hourly space velocity (GHSV) of 9.59 × 10^−4^ L/g s.

Figure 10 shows the model predictions for conversion and product yields as functions of inverse temperature along with the experimental data. The data at very low temperature, 523.15 K, was found to be too low due to loss of activity of the catalyst. Therefore, the model predictions are fitted with straight lines only for the data at higher temperatures (573.15 K and 613.15 K) for determining the kinetic parameters for the 80Fe20Cu/K catalyst. Figure 8 shows significantly higher conversion and yields than in the case without copper present in the catalyst, indicating a larger pre-exponential factor kFT_0_ and kFTs_0_. The kinetic parameters derived by fitting the model predictions (especially C_3_H_6_ and CH_4_ species) to the experimental data shown in Figure 10 are given in Table 4. The FT reaction activation energy was found to be 72 kJ/mol, the same as for the catalyst without copper present (Table 6). The rate constants kFT_0_ and kFTs_0_ for forming C_3_H_6_ and CH_4_ are 5 and 1.5 times higher, respectively, than for the catalyst without copper (Table 6). So, the presence of copper has a significant effect on FT.

## 4. Materials and Methods

### 4.1. Catalyst Synthesis

For each catalyst, potassium nitrate, iron (III) nitrate nonhydrate, and if necessary copper (II) nitrate trihydrate were dissolved in 50 mL of boiling deionized water at the desired ratio. The solution was then added to 20 g of fine gamma alumina powder, stirred with a stir bar to form a paste-like consistency, heated to 90 °C, and allowed to dry. The resulting catalyst material was then calcined overnight at 350 °C and subsequently cooled back down to room temperature. Upon impregnation and calcification, the catalyst material agglomerates and a mortar and pestle were used to achieve an average surface area of 22 m^2^/g. It is worth noting that the surface area of the support calcined without additional metals present has a surface area of 77 m^2^/g and was found not active for CO_2_ hydrogenation. All catalysts synthesized had a compositional mass ratio of 16M:12K:100Al_2_O_3_ (unless otherwise specified), where M includes the total mass of copper and iron components. Different catalyst formulas are denoted by the molar ratio of iron to copper in the catalyst. Potassium and gamma aluminum oxide remain constant throughout all catalysts unless otherwise noted.

### 4.2. Catalytic Activity Measurement

Twenty grams (20 g) of catalyst was packed into the reactor vessel, a ½ inch outer diameter steel tube 12 inches in length. Roughly 10 inches of the tube were packed with catalyst, and glass wool plugs were used at either end to secure the catalyst within the reactor vessel. H_2_, CO_2_, and N_2_ gas flow rates were controlled via three separate mass flow controllers, and the overall pressure of the system was held at 290 psi (~20 bar). The pressure was controlled by a back pressure valve at the end of the system. Temperature was controlled via an aluminum block set around the reactor vessel with four separate heating elements and thermocouples. Water and some liquid oil fractions (C6+) were removed from the gas product stream via a cold trap set at 10 °C. The amount of liquid oil trapped is negligible and does not affect the overall selectivity data. The water component was also checked for alcohols and determined to have a concentration of less than 1 mmol.

All catalysts were reduced under a pure H_2_ gas flow (100 ccm) overnight, after which the input was altered to match our desired CO_2_:H_2_:N_2_ (25:75:10 unless otherwise specified) ratio without any purge or drop in pressure. The flow rate in all experiments was kept to 110 ccm, with a fixed N_2_ rate of 10 ccm for use as an internal standard. The GHSV was 2.6 × 10^−4^ L/s g for all tests. The effluent gases were analyzed in real time using an inline gas chromatograph (GC) (Agilent Technologies, Fast RGA analyzer, Santa Clara, CA, USA). Hydrocarbons were separated using an HP-Al/S column (Agilent Technologies, 19091P-512, 25 μm × 320 μm × 8 μm) and detected with an FID detector. Fixed gases (H_2_, CO_2_, CO, N_2_) were separated on a Unibead IS column (4 ft, 60/80 mesh in UltiMetal) and a 5 Å molecular sieve column (8 ft, 60/80 mesh) and detected with a TCD detector. It is important to note that all selectivites and yields are reported on a per carbon atom consumed basis and not per mole of product (i.e., propane selectivity is weighted by 3 due to the fact that it accounts for three carbon atoms per molecule).

### 4.3. Characterization

Powder X-ray diffraction analysis was performed on a Rigaku Smartlab X-ray Diffractometer using a Cu_K*α* source. A Thermo Scientific K-alpha equipped with a monochromatic Al K*α* source and 180° double focusing hemispherical analyzer with 128-channel detector was used to collect X-ray photoelectron spectroscopy data. SEM measurements were performed with a Carl Zeiss Supra 55 Schottky thermal field emitting microscope using a 30 um aperture at 5–15 KV accelerating voltage. EDS measurements were performed using an Oxford instruments (Abington, UK) 80 mm silicon drift detector, X-Max 80, inside the Supra 55 SEM using an accelerating voltage of 5 kV and working distance of 8.5 mm. The detector was calibrated with a copper standard. Data acquisition was collected on 2048 channels for 500,000 counts using a process time setting of 4 with pulse pile up correction. The quantitative analysis was performed unnormalized using the supplied 5 kV quantitative standard database.

## 5. Conclusions

The synthesis of useful hydrocarbons from carbon dioxide and hydrogen is a matter of interest for scientific study. In order to combat some of the detrimental effect of water on iron-based CO_2_ hydrogenation catalysts, copper was added to the catalyst blend. The addition of copper showed a net gain in CO_2_ hydrogenation activity and decreased the amount of unwanted byproducts significantly. The 90Fe10Cu/K catalyst was determined to be the ratio of copper to iron that yielded the best catalytic activity. The copper does not affect the crystallinity or dispersion of the catalyst, but does create some phase separation effects if the amount of copper added is too high. Copper significantly improves the kinetics of FT reactions, as predicted by the model after accounting for the transport limitations imposed by the particles, the porosity of the bed, and of the catalyst particles consistent with experimental data. The data obtained at different temperatures enable us to derive the activation energy for the FT reaction when copper is included. It was found that the diffusive transport is relatively fast compared to the reaction rate in the particles, and support particle porosity has a significant effect on the conversion and yield.

## Figures and Tables

**Table 1:** Activities of FeCu/K catalysts with various Fe:Cu ratios.

Sample	CO_2_ Conversion (%)	Selectivity (%)			O/P	Alpha	Yield (%)
		CO	Meth	C2–C5			
100Fe/K	24	16	4.6	79	4.29	0.98	19
90Fe10Cu/K	41	5.7	6.4	88	4.88	0.91	36
80Fe20Cu/K	30	6.3	9.7	84	5.52	0.89	25
70Fe30Cu/K	36	5.7	6.0	88	4.06	0.91	32

O/P: olefin/paraffin.

**Table 2:** Effects of K on FeCu/K catalysts.

Sample	CO_2_ Conversion (%)	Selectivity (%)			O/P	Alpha	Yield (%)
		CO	Meth	C2–C5			
Fe80Cu20/K	30	6	10	84	5.5	0.89	25
Fe80Cu20/noK	17	35	11	54	0	0.69	9.3

**Table 3:** Comparison of activities between the Fe90Cu10/K sample, a physical mixture of Fe100/K and Cu100/K, and a layered sample.

Sample	CO_2_ Conversion (%)	Selectivity (%)			O/P	Alpha	Yield (%)
		CO	Meth	C2–C5			
90Fe10Cu/K	38	9.0	4.8	86	4.5	0.95	32
Physical mixture	18	2.8	15	82	0	0.75	15
Layered sample	37	8.1	6.6	85	6.0	0.86	35

**Table 4:** Relationship between temperature and activity for Fe80Cu20/K.

Sample	Temperature (°C)	CO_2_ Conversion (%)	Selectivity (%)			O/P	Alpha	Yield (%)
			CO	Meth	C2–C5			
80Fe20Cu/K	250	12	0	11	89	1.9	0.82	10
	300	30	6.3	9.7	84	5.5	0.89	25
	340	41	8.8	7.9	83	4.5	0.88	34

**Table 5:** Kinetic parameters used for the modeling of 80Fe20Cu/K.

Kinetic Parameter	CO_2_ Shift (SH)	FT	FTs, Methanation
*a_i_*, H_2_O	65 Ref. [31]	33 Ref. [31]	33 Ref. [31]
*b_i_*, CO_2_	7.4 Ref. [31]	2.7 Ref. [31]	2.7 Ref. [31]
*k_i_*_,0_, mol/(s·g·MPa)	10 Ref. [10]	6.45 × 10^3^	7.74 × 10^2^
*K_eq_*_0_	1 Ref. [31]	N/A	N/A
*E*_*A*,*i*_, kJ/mol	55 Ref. [31]	72 Ref. [31]	72 Ref. [31]

FT: Fischer–Tropsch reaction.

**Table 6:** Comparison on kinetic parameters of 100Fe/K and 80Fe20Cu/K.

Sample	*k*_*i*,0_, mol/(s·g·MPa) (FT)	*k*_*i*,0_, mol/(s·g·MPa) (CH_4_)	*E*_*A*,*i*_, kJ/mol (FT)
100Fe/K Ref. [10]	1.29 × 10^3^	5.16 × 10^2^	72
80Fe20Cu/K	6.45 × 10^3^	7.74 × 10^2^	72
